# Investigating public perceptions and knowledge translation priorities to improve water safety for residents with private water supplies: a cross-sectional study in Newfoundland and Labrador

**DOI:** 10.1186/1471-2458-13-1225

**Published:** 2013-12-23

**Authors:** Steven M Roche, Andria Jones-Bitton, Shannon E Majowicz, Katarina D M Pintar, David Allison

**Affiliations:** 1 Department of Population Medicine, University of Guelph, 50 Stone Road East, Guelph, Ontario N1G 2 W1, Canada; 2 School of Public Health and Health Systems, University of Waterloo, 200 University Avenue West, Waterloo, Ontario N2L 3G1, Canada; 3 C-EnterNet Surveillance, Laboratory for Foodborne Zoonoses, Public Health Agency of Canada, 255 Woodlawn Road West, Unit 120, Guelph, Ontario N1H 8J1, Canada; 4 Medical Officer of Health, Eastern Health Region, 760 Topsail Road, Mount Pearl, Newfoundland and Labrador A1N 3J5, Canada

**Keywords:** Knowledge translation, Private water supplies, Public health, Water perceptions, Water testing

## Abstract

**Background:**

The first objective of this study was to investigate the public perceptions of private water and alternative sources with respect to safety, quality, testing and treatment in Newfoundland and Labrador (NL), Canada. The second objective was to provide public health practitioners with recommendations for improving knowledge translation (KT) efforts in NL, based on assessments of respondents’ perceived information needs and preferred KT methods.

**Methods:**

A cross-sectional telephone survey of 618 households with private water supplies was conducted in March-April, 2007. Questions pertained to respondents’ perceptions of their tap water, water concerns, alternative water use, well characteristics, and water testing behaviours.

**Results:**

Approximately 94% of households were supplied by private wells (50% drilled and 50% dug wells), while 6% obtained water from roadside ponds, rivers or springs (RPRS). While 85% rated their water quality highly, 55% nevertheless had concerns about its overall safety. Approximately 11% of respondents never tested their water, and of the 89% that had, 80% tested at frequencies below provincial recommendations for bacterial testing. More than one-third of respondents reported treating their water in the home, and 78% employed active carbon filtration methods. Respondents wanted more information on testing options and advice on effective treatment methods. Targeted advertising through television, flyers/brochures and/or radio is recommended as a first step to increase awareness. More active KT methods involving key stakeholders may be most effective in improving testing and treatment behaviour.

**Conclusions:**

The results presented here can assist public health practitioners in tailoring current KT initiatives to influence well owner stewardship behaviour.

## Background

In Canada, approximately 4 million people rely on private water supplies, primarily groundwater wells [1]. Private water consumers are responsible for maintaining the safety of their water, which includes routine bacterial and chemical testing, which is subsidized in many provinces [2,3]. Recent Canadian reports suggest homeowners test their well water infrequently [1,4-8]. Despite numerous reports that many private water sources in Canada often contain microbes and chemicals at levels above the maximum acceptable concentrations [1,6,9-11], in-home drinking water treatment is uncommon in many rural Canadian homes [1,4]. A lack of knowledge of water quality and safety, coupled with a lack of effective treatment, poses a public health threat [6].

Newfoundland and Labrador (NL) has a population of 505,469; roughly 29% of households rely on public and/or private groundwater sources for their drinking water, with an estimated 40,000 private well owners [12,13].

The first objective of this study was to investigate the public perceptions of water from private supplies and alternative sources in NL with respect to safety, quality, testing and treatment. The second was to identify respondents’ existing information needs and preferred extension methods in an effort to provide public health practitioners with recommendations for improving knowledge translation (KT) efforts in NL.

## Methods

### Study design

A cross-sectional telephone survey of residences served by a private water supply in NL was conducted in March and April, 2007. The sampling frame was developed from a list of telephone exchanges [14], which corresponded to NL community names for private water owners (Government of NL Department of Environment and Conservation (DOEC)). Telephone numbers were then randomly selected using a commercial database, excluding unlisted and ‘do not call’ numbers [15]. Professionally-trained interviewers administered the survey using computer-assisted telephone interviewing.

Given an expectation that 50% of respondents would be concerned about their well water safety, a minimum required sample size of 384, with an allowable error of 5% and 95% confidence and the NL population of 505,469, was exceeded in this study (final sample size = 618).

### Survey methodology

The questionnaire was based on one used in a similar 2004 study in Hamilton, Ontario [5], with question phrasing and definitions of certain words changed slightly based on pre-study focus groups in the NL target population. Open- and closed-ended questions covered respondents’ perceptions of their tap water, water concerns, alternative water use, well characteristics, water testing behaviours, and demographic information. For this study, an alternative water source was defined as any source of water other than the household tap water (i.e. bottled water and treated or untreated water from roadside ponds, rivers or springs (RPRS) consumed in the home) or household tap water that had been treated.

Each interview was conducted in English and took approximately 20 minutes to complete. Calls were made at various times of day and week to reduce non-response bias; four attempts were made to reach each household. The inclusion criteria for participation were: 18 years of age or older and living in a residence with a private water source. The Human Investigations Committee, Memorial University of Newfoundland, provided ethics approval.

### Analyses

Descriptive analyses were performed in STATA/IC 11.2 for Mac (StataCorp). Demographic characteristics of survey respondents were compared to the NL census population [13] using Chi-square tests (*p* < 0.05). Pearson correlation coefficients (r) were calculated to assess the correlation between respondent ratings of the organoleptic properties of their water and their perceptions on water safety.

## Results

Of the 5743 phone numbers contacted, 3022 numbers were eligible (i.e. were not invalid, business or fax lines). Six hundred eighteen surveys were completed, yielding a response rate of 20%. Not all questions were fully answered by all respondents; hence, some analyses were conducted with smaller sample sizes, as noted. Survey respondents were more likely to be female, older and more educated than the general NL population (Table 1).

**Table 1:** Comparison of demographic characteristics between survey respondents (n = 618; collected March-April 2007) and residents of Newfoundland & Labrador (Statistics Canada, 2006)

**Variable**		**Study population # (%)**	**Census population # (%)**	**Chi-square statistic**
Gender (p < 0.0001)	Male	243 (39.3)	245 735 (48.6)	
	Female	375 (60.7)	259 735 (51.4)	21.4
Age (years) (p < 0.0001)	20 to 29*	14 (2.3)	58 615 (14.9)	
	30 to 39	87 (14.1)	67 475 (17.2)	
	40 to 49	145 (23.5)	84 440 (21.5)	
	50 to 59	174 (28.1)	82 175 (20.9)	
	60 to 69	136 (22.0)	52 320 (13.3)	
	>70	59 (9.5)	48 110 (12.2)	
	Unknown	3 (0.5)		125.6
Education level (p < 0.0001)	Grade school	173 (28.0)	141 575 (34.4)	
	High school graduate	193 (31.2)	93 300 (22.6)	
	College/technical school graduate	155 (25.1)	125 480 (30.5)	
	University graduate	67 (10.8)	47 690 (11.6)	
	Post-graduate degree	17 (2.8)	3 615 (0.9)	
	Other	7 (1.1)		
	Unknown	6 (1.0)		59.0
Household income ($ CAD) (p < 0.0001)	< $10,000	26 (4.2)	9 690 (4.9)	
	$10,000 to $14,999	35 (5.7)	12 465 (6.3)	
	$15,000 to $19,999	44 (7.1)	15 015 (7.6)	
	$20,000 to $29,999	81 (13.1)	26 985 (13.7)	
	$30,000 to $39,999	95 (15.4)	25 050 (12.7)	
	$40,000 to $49,999	58 (9.4)	21 190 (10.8)	
	$50,000 to $59,999	45 (7.3)	18 970 (9.6)	
	$60,000 to $69,999	23 (3.7)	15 005 (7.6)	
	> $70,000	82 (13.3)	52 810 (26.8)	
	Unknown	129 (20.9)		46.6
Number of people in household (p < 0.0001)	1	82 (13.3)	39 830 (20.2)	
	2	271 (44.1)	73 295 (37.2)	
	3	124 (20.2)	39 835 (20.2)	
	4	112 (18.2)	31 985 (16.2)	
	5	17 (2.8)	9 370 (4.8)	
	6+	9 (1.4)	2 875 (1.5)	28.8
Mean number of people in household		2.6	2.5	

*Age range of comparison groups differ; census: 20 – 29 years versus sample: 18 – 29 years.

### Household water sources

Approximately 94% (579/618; 95% CI: 91.5-95.4) of households were supplied with drinking water by private wells, 6% (35/618; 95% CI: 4.1-7.8) obtained household tap water from RPRS, and 4 respondents did not know their tap water source.

Of households with a private well, 50% (287/579; 95% CI: 45.5-53.6) were drilled. Among these respondents, approximately 99% (283/287; 95% CI: 96.5-99.5) reported owning a well cover and 89% (214/240; 95% CI: 84.6-92.5) reported having well with a liner. Seventy-four percent (211/287; 95% CI: 68.1-78.3) of respondents reported the depth of their drilled well. The reported depths ranged from 28 to 1200 feet, with an average well depth of 190 feet and a standard deviation of 132 feet.

The other 50% (292/579; 95% CI: 46.4-54.5) of respondents who owned private wells specifically owned dug wells. Among these respondents, approximately 99% (288/292; 95% CI: 96.5-99.5) reported owning a well cover and 72% (193/269; 95% CI: 66.1-76.8) reported having a well with a liner. Seventy-six percent (223/292; 95% CI: 71.2-80.9) of respondents reported the depth of their dug well. The reported depths ranged from 2 to 60 feet, with an average well depth of 12 feet and a standard deviation of 7.5 feet. Additionally, 57% (127/223; 95% CI: 50.4-63.3) of respondents reported having dug wells more shallow than the provincial recommendations for dug well depth (minimum of 12 feet deep [16]).

### Perceptions of drinking water

Among drilled well owners, the majority of respondents rated the taste (94.4%; 268/284; 95% CI: 91.1-96.5), smell (92.3%; 264/286; 95% CI: 88.6-94.9), colour (98.3%; 282/287; 95% CI: 96.0-99.3), and clarity (97.9%; 281/287; 95% CI: 95.5-99.0) of their water as being ‘good’/‘very good’. In addition, 88% (252/285; 95% CI: 84.2-91.6) of respondents were ‘sure’/‘very sure’ that the water from their drilled well was safe to drink. However, 51% (146/286; 95% CI: 45.3-56.8) still reported being ‘concerned’/’very concerned’ about the overall safety of the water they consume from their drilled well.

Among dug well owners, the majority of respondents rated the taste (92.7%; 267/288; 95% CI: 89.1-95.2), smell (94.8%; 276/291; 95% CI: 91.7-96.9), colour (94.5%; 276/292; 95% CI: 91.3-96.6), and clarity (92.8%; 271/292; 95% CI: 89.3-95.3) of their water as being ‘good’/‘very good’. In addition, 84% (244/292; 95% CI: 78.9-87.4) of respondents were ‘sure’/‘very sure’ that the water from their dug well was safe to drink. However, 56% (164/291; 95% CI: 50.6-61.9) still reported being ‘concerned’/’very concerned’ about the overall safety of the water they consume from their dug well. Differences between these perceptions among drilled and dug well owners were not statistically significant (*p* > 0.05).

Among those that used water from RPRS, the majority of respondents rated the taste (82.4%; 28/34; 95% CI: 66.5-91.7), smell (94.1%; 32/34; 95% CI: 80.9-87.4), colour (85.7%; 30/35; 95% CI: 70.6-93.7), and clarity (94.1%; 32/34; 95% CI: 80.9-98.4) of their water as being ‘good’/‘very good’. In addition, 66% (23/35; 95% CI: 49.2-79.2) of respondents were ‘sure’/‘very sure’ that the water from their RPRS was safe to drink. However, 74% (26/35; 95% CI: 57.9-85.8) still reported being ‘concerned’/’very concerned’ about the overall safety of the water they consumed from RPRS.

Respondent ratings on the quality of the organoleptic properties of their water (i.e. taste, smell, colour, clarity), and how sure they were that their water was safe to drink, were correlated (r > 0.5; *p* < 0.05). Of the 84.8% (519/612; 95% CI: 81.7-87.4) of total respondents that were ‘sure’/‘very sure’ that their water was safe to drink, the majority rated the taste (97.5%; 506/519; 95% CI: 95.8-98.5), smell (96.7%; 502/519; 95% CI: 94.8-97.8), colour (98.8%; 513/519; 95% CI: 97.5-99.5), and clarity (97.9%; 508/519; 95% CI: 96.3-98.8) of their water as being ‘good’/‘very good’. Respondents’ level of concern regarding the safety of their water was not well correlated (r < 0.1; *p* < 0.05) with respondent ratings on the quality of the organoleptic properties of their water. Respondents provided open-ended explanations for being ‘concerned’/‘very concerned’ about the overall safety of their water. The majority of responses expressed general health concerns with regard to the long-term safety and quality of their water, rather than immediate concerns regarding their water’s safety (data not shown).

### Water treatment & reasons for use of alternative sources

Forty-one percent (118/285; 95% CI: 35.8-47.2) of respondents owning drilled wells reported treating their drinking water using an in-home treatment method, and 25% (29/118; 95% CI: 17.7-33.1) of these used more than one treatment method.

Thirty-six percent (105/292; 95% CI: 30.7-41.6) of respondents owning dug wells reported treating their drinking water using an in-home treatment method, and 30% (32/105; 95% CI: 22.5-38.8) of these used more than one treatment method. Among the 57% (127/223; 95% CI: 50.4-63.3) of respondents with dug wells more shallow than minimum recommended dug well depth [16], 35% (44/127; 95% CI: 26.9-43.3) reported treating their drinking water using an in-home treatment method.

Twenty-three percent (8/35; 95% CI: 12.1-39.0) of respondents obtaining household tap water from RPRS reported treating their drinking water using an in-home treatment method.

**Figure 1:**
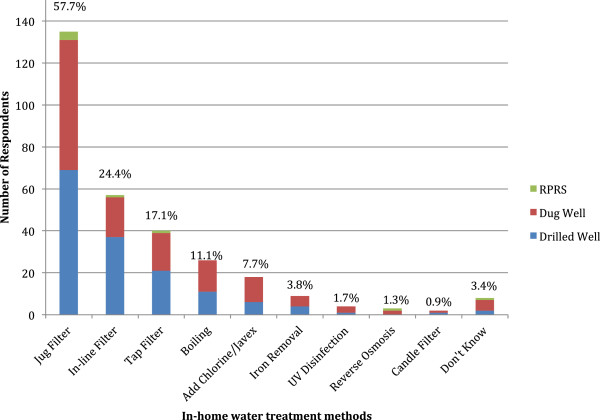
Histogram of the type of in-home drinking water treatment methods employed among households receiving drinking water from drilled wells, dug wells and RPRS, Newfoundland & Labrador, 2007 (n = 234; multiple treatment methods per household permitted).

Briefly, 78% (90/115; 95% CI: 69.9-84.8), 79% (81/102; 95% CI: 70.6-86.1) and 63% (5/8; 95% CI: 30.6-86.3) of respondents with drilled wells, dug wells and water from RPRS reported the use of point-source treatment methods (e.g. jug and tap filters), respectively. Furthermore, 83% (35/42; 95% CI: 69.4-91.7) of respondents with dug wells more shallow than DOEC recommendations reported employing the use of point-source treatment methods. The specific methods employed among respondents with drilled wells, dug wells and water from RPRS are shown (Figure 1).

Twenty-nine percent (177/614; 95% CI: 25.4-32.5) of respondents reported occasionally consuming bottled water instead of the tap water from their private water source whilst at home. Similarly, 5% (26/579; 95% CI: 3.1-6.5) of respondents reported occasionally consuming water from RPRS instead of the tap water from their private water source whilst at home. The proportion of respondents for whom specific factors were ‘important’/‘very important’ in deciding to use water from alternative sources instead of water straight from the household tap are listed (Table 2).

**Table 2:** Proportion of respondents with drilled wells, dug wells or RPRS for whom specific factors were ‘important’/‘very important’ in deciding to use in-home water treatment methods and/or occasionally consume water from alternative sources instead of household tap water, Newfoundland and Labrador, 2007

**Factors important & very important**	**Among drilled well owners # (%; 95% CI)**	**Among dug well owners # (%; 95% CI)**	**Among RPRS consumer # (%; 95% CI)**
**Water treatment devices**	**n = 118**	**n = 105**	**n = 8**
Improved taste	78 (66.1; 57.2-74.0)	75 (71.4; 62.2-79.2)	5 (62.5; 30.6-86.3)
Improved smell	70 (59.3; 50.3-67.7)	67 (63.8; 54.3-72.4)	6 (75.0; 40.9-92.9)
Reduced germs/bacteria/E. coli	92 (78.0; 69.7-84.5)	89 (84.8; 76.7-90.4)	6 (75.0; 40.9-92.9)
Reduced metals or minerals	92 (78.0; 69.7-84.5)	74 (70.5; 61.2-78.4)	7 (87.5; 52.9-97.8)
Reduced chemicals	88 (74.6; 66.0-81.6)	76 (72.4; 63.2-80.0)	6 (75.0; 40.9-92.9)
Reduced cloudiness	76 (64.4; 55.4-72.5)	70 (66.7; 57.2-75.0)	6 (75.0; 40.9-92.9)
Reduced hardness	61 (51.7; 42.8-60.5)	55 (52.4; 42.9-61.7)	6 (75.0; 40.9-92.9)
**Bottled water**	**n = 79**	**n = 88**	**n = 9**
Improved taste	48 (60.8; 49.7-70.8)	57 (64.8; 54.4-73.9)	5 (55.6; 26.7-81.1)
Improved smell	47 (59.5; 48.5-69.6)	53 (60.2; 49.8-69.8)	3 (33.3; 12.1-64.6)
Reduced germs/bacteria/E. coli	51 (64.6; 53.6-74.2)	64 (72.7; 62.6-80.9)	7 (77.8; 45.3-93.7)
Reduced metals or minerals	52 (65.8; 54.9-75.3)	59 (67.0; 56.7-76.0)	5 (55.6; 26.7-81.1)
Reduced chemicals	50 (63.3; 52.3-73.1)	59 (67.0; 56.7-76.0)	5 (55.6; 26.7-81.1)
Reduced cloudiness	47 (59.5; 48.5-69.6)	52 (59.1; 48.7-68.8)	6 (66.7; 35.4-87.9)
Reduced hardness	31 (39.2; 29.2-30.3)	48 (54.5; 44.2-64.5)	3 (33.3; 12.1-64.6)
Better safety testing/control	52 (65.8; 54.9-75.3)	61 (69.3; 59.0-78.0)	6 (66.7; 35.4-87.9)
Convenience	49 (62.0; 51.0-71.9)	58 (65.9; 55.5-75.0)	3 (33.3; 12.1-64.6)
**Water from RPRS**	**n = 8**	**n = 17**	NA*
Improved taste	6 (75.0; 40.9-92.9)	15 (88.2; 65.7-96.7)	-
Improved smell	6 (75.0; 40.9-92.9)	15 (88.2; 65.7-96.7)	-
Reduced germs/bacteria/E. coli	6 (75.0; 40.9-92.9)	16 (94.1; 73.0-99.0)	-
Reduced metals or minerals	5 (62.5; 30.6-86.3)	16 (94.1; 73.0-99.0)	-
Reduced chemicals	5 (62.5; 30.6-86.3)	15 (88.2; 65.7-96.7)	-
Reduced cloudiness	7 (87.5; 52.9-97.8)	13 (76.5; 52.7-90.4)	-
Reduced hardness	5 (62.5; 30.6-86.3)	11 (64.7; 41.3-82.7)	-
Convenience	0 (0.0; 0.0-32.44)	0 (0.0; 0.0-18.4)	-

*RPRS are the household tap water source for these respondents and thus are omitted here.

### Water testing

**Figure 2:**
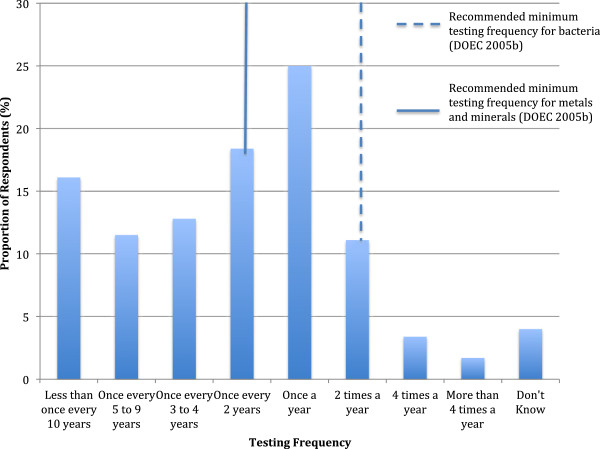
Frequency with which 613 respondents reported testing the water from their private source, Newfoundland & Labrador, 2007.

Eleven percent (68/613; 95% CI: 8.8-13.8) of respondents reported never having tested their water. Of the 89% (545/613; 95% CI: 86.2-91.2) that had tested at some point in the past, 80% (438/545; 95% CI: 67.8-74.9) reported testing at frequencies below the provincial recommendations (Figure 2) [17]. More specifically, 86% (209/244; 95% CI: 80.7-89.5), 83% (212/255; 95% CI: 78.1-87.2) and 81% (17/21; 95% CI: 60.0-92.3) of respondents with drilled wells, dug wells and water from RPRS reported testing at frequencies below the provincial recommendations, respectively [17]. Among those dug well owners with wells more shallow than DOEC recommendations, 88% (100/113; 95% CI: 81.3-92.3) reported testing at frequencies below the provincial recommendations. Explanations for not testing water at the recommended frequency are shown (Table 3).

**Table 3:** Respondent explanations for not testing their private water supplies at a frequency that met or exceeded provincial recommendations, Newfoundland & Labrador, 2007 (n = 339; multiple explanations per respondent permitted)

**Explanation**	**Frequency # (%)**
No noticeable changes to water, looks and smells normal, trust it*	93 (27.4)
No problems noted in general*	41 (12.1)
Inconvenient to drop off a sample for testing	33 (9.7)
There is no need to test the water that frequently*	30 (8.8)
Inconvenient to pick up sample bottle	25 (7.4)
Lack of information on testing	20 (5.9)
Previous test results were normal*	17 (5.0)
Don’t drink water from the private water source	15 (4.4)
Inconvenient (in general)*	14 (4.1)
No particular reason*	14 (4.1)
Forget or procrastinate*	8 (2.4)
No health problems among household members noted*	6 (1.8)
Others nearby test their water and results normal, so no need*	5 (1.5)
Use a water treatment system*	5 (1.5)
Misinformed about recommended testing frequency*	3 (0.9)
Plan to test soon*	3 (0.9)
Cost*	2 (0.6)
Other**	5 (1.5)

*Initially coded as “other” in closed-ended question; re-categorized based on responses to a follow-up open-ended question.

**Verbatim other responses include:

I was getting the same answer that the water wasn’t suitable for drinking.

We tried to get it tested but the Government wouldn’t do it.

I just moved in.

Afraid they might say it’s not safe to drink.

Inaccurate test results in the past make you question the reliability of the testing.

In total, 59% (360/612; 95% CI: 54.9-62.7) of respondents were aware that the NL government offered free bacterial testing of water from private water supplies. Of those aware of the service, 84% (303/360; 95% CI: 80.0-87.6) had used it in the past, 97% (295/303; 95% CI: 94.9-98.7) of which were satisfied with this service. Of the 11% (68/613; 95% CI: 8.8-13.8) of respondents that reported never having tested their water, 71% (48/68; 95% CI: 59.8-80.1) were unaware of this free testing. Similarly, of the 86%, 83% and 81% of respondents with drilled wells, dug wells and water from RPRS that tested at frequencies below provincial recommendations, 39% (112/284; 95% CI: 33.9-45.2), 41% (119/289; 95% CI: 35.7-46.9) and 51% (18/35; 95% CI: 35.6-67.0) were unaware of the free testing, respectively.

Of those who tested their water in the past, 54% (296/545; 95% CI: 50.1-58.5) tested for *E. coli* and fecal coliforms. Testing for other contaminants, including other bacteria (25%; 135/545; 95% CI: 21.3-28.6), heavy metals (29%; 160/545; 95% CI: 25.7-33.3), pesticides (21%; 112/545; 95% CI: 17.4-24.1), sodium (20%; 107/545; 95% CI: 16.5-23.2) and nitrates (19%; 103/545; 95% CI: 15.8-22.4) was less common.

### Public education

Eighty-four percent (519/616; 95% CI: 81.2-86.9) of respondents reported it was ‘important’/‘very important’ they receive more information on private water source testing (e.g. where it can be done, how often it should be done, what tests are available, what parameters should be tested). Sixty-four percent (387/601; 95% CI: 60.5-68.1) reported it was ‘important’/‘very important’ they receive water test results from other private sources in their area. Seventy-eight percent (474/605; 95% CI: 74.9-81.4) reported it was ‘important’/‘very important’ they receive more advice on water treatment options.

Respondents indicated how likely they would be to use various KT methods to access pertinent water information, and which methods they considered would be most effective in advertising DOEC’s free bacterial testing (Table 4).

**Table 4:** Proportion of respondents who reported the likelihood that they would utilize various knowledge translation methods to access water testing and treatment information pertaining to their private water source(s), and where they felt the province could best advertise the free bacterial testing service, Newfoundland & Labrador, 2007

**Media source/outlet**	**Very likely # (%; 95% CI)**	**Likely # (%; 95% CI)**	**Neither likely nor unlikely # (%; 95% CI)**	**Unlikely # (%; 95% CI)**	**Very unlikely # (%; 95% CI)**	Preferred advertisement method* # (%; 95% CI)
Television (n = 616)	221 (35.9; 32.2-39.7)	294 (47.7; 43.8-51.7)	8 (1.3; 0.7-2.5)	54 (8.8; 6.8-11.3)	39 (6.3; 4.7-8.5)	356 (64.6; 60.5-68.5)
Flyer/brochure (n = 617)	279 (45.2; 41.3-49.2)	242 (39.2; 35.5-43.1)	8 (1.3; 0.7-2.5)	53 (8.6; 6.6-11.1)	35 (5.7; 4.1-7.8)	224 (40.7; 36.6-44.8)
Newspaper (n = 613)	168 (27.4; 24.0-31.1)	259 (42.3; 38.4-46.2)	12 (1.9; 1.2-3.6)	116 (18.9; 12.9-18.6)	58 (9.5; 6.0-10.2)	220 (39.9; 35.9-44.1)
Radio (n = 610)	185 (30.3; 26.8-34.1)	269 (44.1; 40.2-48.1)	13 (2.1; 1.3-3.6)	95 (15.6; 12.9-18.7)	48 (7.9; 6.0-10.3)	206 (37.4; 33.5-41.5)
NL government website (n = 609)	113 (18.6; 15.7-21.8)	166 (27.3; 23.9-30.9)	10 (1.6; 0.9-3.0)	159 (26.1; 22.8-29.7)	161 (26.4; 23.1-30.1)	30 (5.4; 3.8-7.7)

*Data shown here collected in separate question (n = 551; multiple responses per respondent permitted.

Respondents identified a variety of methods they thought might help increase water testing in NL, including: water sample pickup directly from residences (72.1%; 444/615; 95% CI: 68.5-75.6), reminder pamphlets (67.6%; 416/615; 95% CI: 63.9-71.2), water collection bottle drop-off at residences (65.7%; 404/615; 95% CI: 61.9-69.3), sample drop-off at nearby community centres (62.1%; 382/615; 95% CI: 58.2-65.9), increasing the number of drop-off locations (62.0%; 381/615; 95% CI: 58.1-65.7), and reminders in newspapers (48.9%; 301/615; 95% CI: 45.0-52.9).

## Discussion

The risk of well water contamination differs depending on well type (i.e. drilled vs. dug). Roughly half of survey respondents in the present study reported owning a drilled well, while the other half owned dug wells. Upon comparison, a similar proportion of owners reported owning a well cover (drilled: 99%, dug: 99%), while a higher proportion of drilled owners reported having a well liner (drilled: 89%, dug: 71%). The average depth of drilled wells in this study was similar to that reported by the provincial government [18]. However, nearly 60% of dug well owners reported depths that were shallower than the minimum recommended depths by the provincial government [16]. Dug wells (generally between 12 and 20 feet deep) are at higher risk of contamination than drilled wells (typically 150 feet deep), as water closer to the surface, obtained from shallow groundwater aquifers, is more likely to be contaminated from above-ground sources (e.g. livestock waste, fuel, pesticides) [18]. Sarkar *et al.* (2012) reported that 63% and 10% of summer samples from dug wells in western NL tested positive for total coliforms and fecal coliforms above acceptable limits, respectively, while only 23% of summer samples from drilled wells tested positive for total coliforms above acceptable limits; no summer samples from drilled wells tested positive for fecal coliforms. Additionally, they reported that 80% and 10% of fall samples from dug wells tested positive for total coliforms and fecal coliforms above acceptable limits, respectively, while only 28% of fall samples from drilled wells tested positive for total coliforms above acceptable limits; no fall samples from drilled wells tested positive for fecal coliforms [8]. Given this evidence, a significant proportion of dug well owners in this study can be classified as having a higher risk of well water contamination due to well depth alone. Public health officials must be diligent in raising awareness of the risks of shallow water wells and ensuring that owners of higher risk wells are aware of the testing and treatment options to maintain water quality and safety, if reconstructing the well is not an option.

More than three quarters of well owners, and two thirds of RPRS consumers, reported that they were ‘sure’/’very sure’ that the water they consumed in the home was safe to drink. Furthermore, regardless of household tap water source (e.g. drilled wells, dug wells, RPRS) the majority of respondents rated the organoleptic properties of their household tap water as ‘good’/‘very good’, and these ratings were correlated with perceived water safety. Other studies have identified similar relationships, suggesting that individual perceptions of water safety are primarily influenced by organoleptic properties of water [19,20]. Despite a high proportion of respondents perceiving their water as safe to drink, more than half of the well owners, and three quarters of RPRS consumers, reported that they were ‘concerned’/’very concerned’ about the overall safety of their water. Similar findings have been found among private water users in Hamilton, Ontario where an even larger discrepancy between respondent ratings of safety and level of concern were observed [5]. Jones *et al.* (2006a) suggests that this seemingly contradictory evidence may be due to a lack of perceived health problems from consuming water. We hypothesize that the levels of concern evidenced in this study represent a general level of concern, or interest, in the safety of the water they consume, rather than an immediate, specific concern over its safety. The numerous water-borne disease outbreaks reported in Canada [21-23] in recent years may contribute to this level of concern. Therefore, whilst consumers perceive their water as being safe and of high quality, it might still be expected that consumers will exhibit strong concerns with respect to the potential for threats to the safety of their water and how it may impact their health.

Treatment of water from private sources is an important and effective part of the multi-barrier approach to ensuring water quality and safety [24]. More than one-third of respondents reported treating the water from their private source for drinking. Perceived improvements in the sensory characteristics of water and reduced concentrations of contaminants were the main drivers for treating water in the home among drilled and dug well owners, as well as those who obtained household tap water from RPRS. A variety of treatment methods were reported, with more than a quarter of respondents with drilled and dug wells using more than one method. Similar to several studies on households with municipal systems [25-27], more than two-thirds of respondents reported the use of devices that employ active carbon filtration (i.e. jug and tap filters). However, the efficacy of these treatment methods for private water supplies is questionable as manufacturers recommend they be used only with municipally treated or microbiologically safe water [1]. A recent study in the western region of NL examined changes in water quality among samples from privately owned sources taken before and after treatment using common in-home treatment methods (i.e. water softener, sediment, fridge and carbon filters) and concluded that the treatment methods were largely ineffective at providing clean/safe water [8]. In the current study, only 35% of dug well owners, at higher risk of water contamination due to inadequate well depth (< 12 feet deep), reported treating their water and over 80% of them reported the use of these point-source/active carbon filtration treatment methods, which, as evidenced, may be an inadequate form of treatment. While it is important to note that certain water sources represent different levels of risk (deeper wells less so than shallower wells or water from RPRS), and thus require different levels of treatment, the use of these devices may therefore represent a false sense of security to the homeowner. These potential inefficiencies should be highlighted in future educational materials, with an emphasis on more effective methods of treatment available (e.g. ultraviolet disinfection systems) for those who have water at higher risk of contamination. Additionally, more awareness should be generated surrounding the efficacy and need for water treatment devices, as certain individuals may or may not require sophisticated treatment devices depending on the quality of their water.

Nearly 30% percent of respondents reported occasionally consuming bottled water at home, instead of water from their private source, largely due to perceived improvements in safety and quality control, and reduced concentrations of contaminants. Similar to a previous Canadian study [5], while the perceived convenience of bottled water was a contributing factor, it was not a significant one, as individuals reported on water consumption in the home, limiting the likelihood that convenience would be a significant driver.

A small proportion of respondents reported obtaining their household tap water from RPRS. Additionally, a small proportion of well owners reported occasionally consuming water from RPRS instead of water from their private source while at home, largely due to perceived improvements in taste, smell and reduced concentrations of contaminants. This is an important public health consideration as water from such sources represents a different set of risks to consumers. A recent study assessing bacteriological quality of RPRS in NL reported that 24% of samples were above maximum acceptable limits for *E. coli* or coliforms [28]. The DOEC states that these surface water sources are unsafe due to rapid changes in quality and infrequent testing, and highlights that whilst boiling water from these sources may kill microbes, it is ineffective in removing harmful chemicals, and should not be consumed [29]. Water from RPRS therefore represents a unique and important set of risks to consumers when compared to consumption of water from other sources. Currently, published literature on consumption of water from RPRS is sparse, providing little data on current consumption patterns in Canadian communities. The authors recommend that future studies investigate consumption patterns and factors associated with consumption from this source in an effort to support the creation of risk assessments, which can help direct public health initiatives in the future.

Currently, the DOEC recommends that private well owners test their water for bacteria at least twice a year and for metals/minerals, every two years [17]. Routine water quality monitoring for water from private water sources is not only recommended by Health Canada, it is recommended by every province in Canada [30]. Monitoring data can be helpful in providing residents with a snapshot of their water quality and provides background data on the quality of their water, which they can use as a benchmark to monitor changes in quality over time. Routine testing and monitoring is part of an effective multi-barrier approach to ensuring water quality and safety [24]. Nearly 90% of respondents reported testing their water at least once in the past; however, only 20% of those individuals met the provincial guidelines for testing [17]. Similarly, among those dug well owners, at higher risk of water contamination due to shallow well depth (< 12 feet deep), only 12% reported testing their water at the provincially recommended frequencies. These results are similar to other Canadian reports, which indicate that rural residents test their drinking water infrequently, if at all [1,4,5,8].

Two common explanations for not testing more frequently here included: no noticeable changes to organoleptic properties of water and no obvious health problems among household members. A recent study of private well owners in Newfoundland reported similar reasons for respondents having full faith in their water quality; however, upon subsequent testing of the respondents’ water, more than half of the samples had aesthetic and contaminant parameters higher than acceptable limits [8]. While testing when changes in sensory characteristics of water are noticed is certainly recommended, it is important to note that changes in organoleptic properties do not always occur with chemical contaminants, such as nitrates and pesticides [31]. Future education initiatives must continue to emphasize the need for routine water quality monitoring, as assessing the organoleptic properties of water is not sufficient [5]. Thus, monitoring must be recommended through a multi-barrier approach that involves regular water testing and monitoring changes in organoleptic properties.

Another common explanation for not testing more frequently among respondents in this study was the belief that there is no need to test that frequently. However, water contamination can occur intermittently and is affected by a variety of anthropogenic and environmental factors [31-33]. Increasing awareness about the reasons for regular testing of water from private sources must continue to be a priority for public health units to ensure well owners are aware of the quality and safety of their drinking water, and the potential threats to it.

Similar to a previous Canadian study [5], more than 40% of respondents were unaware of the free bacterial testing offered by the provincial government. Forty percent of the individuals that tested below the provincial guidelines, and 70% of those that had never tested, were unaware of the service. Of the respondents that were aware, over 80% had used the free service. Thus, it seems reasonable to expect that increased awareness of the bacterial testing service would increase the number of individuals submitting samples for testing. Respondents also indicated that they felt the service could be best advertised through television ads or mail-out flyers/brochures; future awareness campaigns should consider using these dissemination methods in order to increase local awareness. In addition, future studies may benefit from assessing the ways in which individuals with a certain background level of knowledge about water quality became informed about current water quality initiatives in their areas. Probing the perceptions and behaviours of ‘informed’ individuals may provide further insights into the use and efficacy of existing KT channels.

Respondents indicated that steps in the testing process were inconvenient (e.g. inconvenient to pick-up collection bottles or drop-off samples at a public health unit) and presented a barrier to more frequent water testing. A similar Canadian study in southern Ontario concluded that inconvenience and a lack of time were significant barriers to routine private well water sampling [34]. Furthermore, an Ontario study reported that inconvenience was a relatively minor factor among owners that regularly tested, as they perceived testing to be important; however, those who did not test on a regular basis saw the inconvenience of testing as a more significant constraint, as they were relatively complacent about testing [35]. The motivation of an individual to test their water for chemical or bacterial safety is dependent on a number of factors (e.g. perceptions, attitudes, previous experiences) and these factors interact to influence an individual’s decision. If the perceived inconvenience of the testing process can be removed or lessened, those who feel that testing is important, yet inconvenient, may be more inclined to submit a water sample. Public health officials and relevant stakeholders should examine the steps of the testing process and consider ways in which the process can be streamlined for the public. More than half of the respondents in this study felt that making sample bottle pick-up and drop-off more convenient would increase testing. Although providing a door-to-door testing process may be resource intensive, the authors recommend that various steps in the testing process could be evaluated to identify opportunities to make sample bottles more accessible to the public (i.e. increase the number and vary the locations of sample bottle collection pick-up/drop-off sites).

The majority of respondents emphasized that it was ‘important’/’very important’ they receive more information on testing and treatment options. Interestingly, the majority of this wanted information is already available on the DOEC website. In a recent study, results from interviews with key stakeholders across Canada indicated that, while the internet provides a cost-effective and easily updatable delivery method, a preference for other delivery methods, lack of access to communication technology, and digital illiteracy among private well owners remain significant barriers to information uptake via internet [36]. Furthermore, they suggest that internet content should be presented in a variety of other formats, including hard copy, telephone, CD-ROM and USB, to meet end-user needs [36]. Internet accessibility did not seem to be the primary reason for the results here, as 62% of respondents reported having internet access. However, when asked how likely respondents would be to use the website to access needed information, over 50% of respondents reported it was ‘unlikely’/‘very unlikely’, and nearly 95% felt that advertising testing services through the website was ineffective. Furthermore, over 75% of respondents reported they would be ‘likely’/‘very likely’ to access information through mail-out flyers/brochures, television and radio advertisements. This evidence provides strong support for the creation and implementation of additional KT methods to disseminate needed information to residents with private water sources.

Typically, KT initiatives aimed at increasing awareness and promoting changes in stewardship behaviour among private well owners have focused on the creation of concise and practical educational materials highlighting ‘best management practices’ for well owners. While making these materials more reader-friendly and practical to use is an important step in the KT process, these materials alone are not likely to create substantial behaviour change [35,37]. Behavioural change research suggests that there is no clear causal relationship between providing information and changing behaviour [37]. Furthermore, health education theories, such as the Health Belief Model, suggest that a set of constructs (i.e. perceived seriousness, perceived susceptibility, perceived benefits and perceived barriers) and modifiers (e.g. education level, motivation) have a major impact on an individual’s willingness to change a behavior [38]. Trade-offs between perceived risk and threat of illness and costs and/or time to test are barriers health practitioners must consider. Additional factors, such as the perceived value in treating and testing water and individual motivation must be addressed through outreach initiatives in order for the educational materials to be effectively used to facilitate a change in behaviour.

The dissemination of educational resources to well owners in a more targeted fashion (i.e. mail-out flyers, television ads, radio ads) may be more effective than the more passive methods being currently used (i.e. internet). In doing so, individuals that are motivated to change, but still require additional information to make an informed decision, will start receiving the information they require, which in turn may result in a water sample being submitted. Additionally, a more targeted dissemination approach may motivate certain individuals who may have been previously unaware of well testing information, in part due to the passive methods currently being employed. Removing key barriers, such as an inconvenient testing process, and ensuring the use of more deliberate dissemination methods, will be important steps in improving the stewardship behaviour of NL well owners.

More active, in-depth KT approaches that involve key stakeholders directly may be the most effective method to achieve desired behaviour change among private well owners. A recent study in Rhode Island implemented and evaluated a voluntary, active-learning process that utilizes community-based workshops with well owners and a variety of other stakeholders (i.e. university extension personnel, health officials) to motivate private well owners to test and protect their drinking water. Upon evaluation of this process, they concluded that structured, one-time workshops were effective in influencing behaviour change, and further reported that 87% of participants contacted health officials to follow-up and obtain additional information [39]. Similarly, Clemens *et al.* (2007) created the ‘Master Well Owner Network’, which used a series of workshops to train volunteers who then helped educate rural residents about private water system management. They concluded that in a one and a half year period, 243 trained volunteers had educated over 7,000 Pennsylvania residents [40]. While active KT approaches such as these can be resource intensive up-front, these approaches have been shown to not only be effective, but also bring together all essential stakeholders in a participatory effort towards improving private well water safety and quality.

The low response rate in this study (20%) may have contributed to selection bias in the form of non-response bias. Based on demographic comparisons, our respondents were more likely to be female, older and more educated than the general population of NL. Given this information, the extent to which our results may be generalized to the broader population may be limited. Provincial data on well characteristics (e.g. liner, cover, etc.) do not currently exist; more information on the physical characteristics of wells in NL is needed in order to evaluate study representativeness from this perspective. Furthermore, given the low response rate, the study sample size lacked the power to assess spatial trends. Geographic or regional differences are important considerations when assessing waterborne disease risk and evaluating drinking water-related resources, perceptions, needs, and behaviours [41]. Future studies should consider regional differences in the characteristics evaluated in this study, as they would highlight those areas in need of resources or posing greater risks to the public, which would strengthen decision-making at the local level.

## Conclusion

While the majority of respondents rated their drinking water as high quality, many were concerned about its safety, and frequent use of water treatment, bottled water, and to a lesser extent, RPRS were reported. To date, there is little literature reporting data on water consumption from RPRS.

There was a clear need for more information on the importance and availability of private water testing and treatment options. While the free bacterial testing service appears to be useful, more targeted advertising through television, flyers/brochures and radio were reported by respondents to be more effective than a website in increasing testing frequency and awareness. Furthermore, more active KT methods involving key stakeholders may be the key to facilitating a change in behaviour towards improving well water stewardship.

The results and recommendations presented here can assist public health practitioners in tailoring their current KT initiatives to be more effective in reaching their target audience and improving well stewardship behaviour. In turn, these measures will help create a more informed community and promote a more proactive approach to preventing and controlling public health issues related to private water supplies.

## Competing interests

The authors declare that they have no competing interests.

## Authors’ contributions

AJB conceived the project and constructed the study design. AJB also supervised the administration of the surveys and collection of the data. SMR carried out preliminary statistical analysis, with all authors contributing to final analyses and interpretations. SMR also drafted the initial manuscript, with all authors providing feedback and critically reviewing the manuscript for publication. All authors read and approved the final manuscript.

## Pre-publication history

The pre-publication history for this paper can be accessed here:

http://www.biomedcentral.com/1471-2458/13/1225/prepub
